# Hemolytic Anemia Leading to Fulminant Hepatic Failure as the Initial Presentation of Wilson’s Disease in a Young Female

**DOI:** 10.7759/cureus.62966

**Published:** 2024-06-23

**Authors:** Mona Ghias, Lindsay Sunzeri, Leslie-Joy Romero, Kevin Bogdansky, Casandra Arevalo Marcano

**Affiliations:** 1 Internal Medicine, West Virginia University, Morgantown, USA; 2 Internal Medicine, West Virginia University School of Medicine, Morgantown, USA; 3 Pediatric Hospital Medicine & Internal Medicine, West Virginia University School of Medicine, Morgantown, USA; 4 Nephrology, West Virginia University School of Medicine, Morgantown, USA; 5 Pulmonary Medicine, West Virginia University School of Medicine, Morgantown, USA

**Keywords:** fulminant wilson disease, wilson disease, acute hepatic failure, hyperbilirubinemia, fulminant hepatic failure, hemolytic anemia

## Abstract

Wilson’s disease (WD) is an autosomal recessive disorder that impairs copper metabolism. Copper accumulates in vital organs such as the brain, liver, and kidneys. The disease typically starts with copper accumulation in the liver and can initially present as acute hepatitis and hepatomegaly. Hemolytic anemia is a typically uncommon complication of WD. We present the case of a healthy 18-year-old female who presented with hemolytic anemia and quickly decompensated to fulminant hepatic failure requiring a liver transplant due to previously undiagnosed WD. This case recognizes the importance of early diagnosis as treatment can be lifesaving.

## Introduction

Wilson’s disease (WD), or hepatolenticular degeneration, is an inherited metabolic disorder of copper metabolism [1]. WD is characterized by the accumulation of copper in various organs such as the liver, brain, and kidneys [1]. As a diagnosis with a large age range on presentation, WD must be a part of the differential diagnosis for patients of varying ages [1]. Neurological and hepatic involvement affects the majority of patients [1]. Severe hemolytic anemia and fulminant hepatic failure as the initial presentation of WD is rare, and the hemolytic anemia can subsequently lead to a delay in diagnosis and initiation of treatment. This presentation of fulminant hepatic failure and hemolytic anemia carries a 95% mortality rate if left untreated, and the only definitive treatment is an emergent liver transplant [1,2].

We present the case of an 18-year-old otherwise healthy female who initially presented with severe and refractory hemolytic anemia. The patient was suspected to have fulminant hepatic failure secondary to WD. She was promptly transferred to a liver transplant-capable facility and underwent an emergent liver transplant. This case highlights the necessity for prompt, accurate diagnosis and recognition, as a delayed diagnosis of WD can lead to decompensated hepatic failure and an increased risk of mortality [3].

## Case presentation

A previously healthy, Caucasian 18-year-old female presented to an emergency department in West Virginia with a five-day history of nausea, vomiting, and right upper quadrant abdominal pain. The patient was noticeably jaundiced and reported dark, cola-colored urine. She had no history of any hospitalizations or medical problems before this admission. Family history was negative for liver disease or other serious illnesses.

Upon presentation, laboratory findings were suggestive of hemolytic anemia with a hemoglobin level of 6.6 g/dL (normal range = 13.4-17.5 g/dL), total bilirubin level of 15.7 mg/dL (normal range = 0.3-1.2 mg/dL), and direct bilirubin of 8.2 mg/dL (normal range = 0.1-0.4 mg/dL). Lactate dehydrogenase (LDH) was mildly elevated at 307 U/L (normal range = 135-225 U/L), reticulocyte count was elevated, and haptoglobin was low. The patient had a negative Coombs test.

The patient’s transaminases were also elevated. Aspartate aminotransferase (AST) was 118 U/L (normal range = 8-40 U/L), alanine transaminase (ALT) was <5 U/L (normal range = 4-36 U/L), and alkaline phosphatase (ALP) levels were significantly low at 18 U/L (normal range = 40-120 U/L). The patient’s international normalized ratio (INR) was notably prolonged at 2.85, suggesting compromised hepatic function (normal ≤1.1).

Within 24 hours of presentation, the patient’s hospital course was complicated by acute mental status changes, including confusion and somnolence. She did not exhibit tremors, Parkinsonism, or dysarthria. This rapid decline in mental status was also associated with worsening laboratory findings during which her total bilirubin levels increased to 45 mg/dL and LDH increased to 836 U/L. Her hemoglobin remained low despite receiving multiple packed red blood cell (pRBC) transfusions.

Test results drawn on admission now revealed a notably high serum copper level at 2.03 mg/dL (normal range = 0.7-1.4 mg/dL) and a low ceruloplasmin level of 17 mg/dL (normal range = 20-100 mg/dL), along with increased urine copper excretion at 11,406 µg/day (normal range = 20-50 µg/day).

The constellation of findings of hemolytic anemia and fulminant hepatic failure, along with specific laboratory markers (low ceruloplasmin, elevated urine copper excretion, and markedly low ALP levels) led to the diagnosis of WD [1].

The diagnosis was further confirmed by analyzing ratios such as the ALP/bilirubin ratio being greater than 4 and the AST/ALT ratio exceeding 2.2. These results strongly indicated that the patient likely had fulminant hepatic failure from WD.

Given the patient’s critical condition and the high potential for life-threatening complications, she was emergently transferred to the nearest transplant-capable facility. She subsequently received a liver transplant four days after being admitted to our facility.

**Figure 1:**
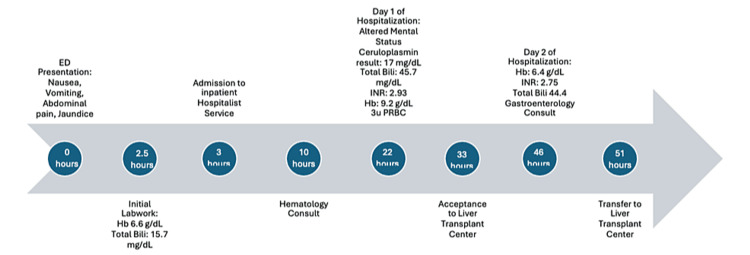
Timeline of hospitalization. ED = emergency department; Hb = hemoglobin; Total Bili = total bilirubin; INR = international normalized ratio; pRBC = packed red blood cell

## Discussion

WD presenting initially as fulminant hepatic failure and severe hemolytic anemia is a rather rare occurrence. However, according to a guideline from the American Association for the Study of Liver Diseases, Coombs-negative hemolytic anemia with features of acute intravascular hemolysis should be considered when the diagnosis of fulminant hepatic failure due to WD is suspected [4]. Most often, a Coombs-negative acute intravascular hemolysis occurs as a consequence of oxidative damage to erythrocytes, which is triggered by high copper concentrations. Patients can also present with dark urine and renal failure. This presentation on the whole carries a grave prognosis and is considered an indication for urgent liver transplantation. Evaluation for transplant would be the only intervention that would essentially stop the hemolysis and provide a cure [4,5].

This case involves a young and otherwise healthy female who showed a severe but non-specific initial manifestation of severe, persistent, Coombs-negative hemolytic anemia. Through quick diagnostic workup and the aid of subspecialty consultants, her treating physicians were able to quickly attribute her acute liver failure and severe persistent hemolytic anemia to WD.

Limitations in the treatment of this patient include her fast medical decompensation into fulminant hepatic failure and delay in laboratory results for copper and ceruloplasmin levels. Her diagnosis was not confirmed until the second day of hospitalization.

Despite the limitations, the patient was transferred to a transplant-capable facility quickly, received a liver transplant, and survived. As of June 2024, the patient is still alive and well.

## Conclusions

This case highlights the importance of keeping WD as a top differential in cases of fulminant and acute hepatic failure, especially in young patients. Hemolytic anemia can also be a presenting feature and can confound and potentially delay diagnosis and treatment due to a patient’s rapidly deteriorating condition. Keeping a high index of suspicion along with prompt serum and urine copper, and ceruloplasmin measurements can lead to early diagnosis and lifesaving intervention such as liver transplantation. This case emphasizes the need for increased awareness among physicians of the varying presentations of WD and the importance of quick diagnosis to prevent rapid patient deterioration.
